# Complete Pectoralis Major Tendon Rupture With Bony Avulsion in an Adolescent Male: A Case Report and Literature Review

**DOI:** 10.7759/cureus.51616

**Published:** 2024-01-03

**Authors:** Kai Zhu, Trisha Vuong, Andrew Pastor, Paul Reynolds

**Affiliations:** 1 Orthopedics, Washington State University Elson S. Floyd College of Medicine, Everett, USA; 2 Orthopedics, The Everett Clinic, Edmonds, USA

**Keywords:** avulsion fracture, pectoralis major tendon rupture, pectoralis major tendon, pectoralis major, pec major

## Abstract

Pectoralis major (PM) tendon ruptures are rare. Typically, they are caused by eccentric contractions from weight lifting. Due to the rarity of pectoralis major tendon ruptures, clinicians might misdiagnose this condition. We report a 16-year-old male with a right pectoralis major tendon rupture and an avulsion fracture after falling on a grass field playing soccer. He was initially misdiagnosed with biceps tendonitis, which highlights the importance of including pectoralis major tendon ruptures in one’s differential diagnoses.

## Introduction

The pectoralis major (PM) is the largest and most superior muscle of the anterior chest wall [1,2]. It is a fan-shaped multi-pennate muscle that has two heads, a clavicular and a sternocostal head [3]. The clavicular head originates from the medial clavicle, while the sternocostal head originates from the sternum [3]. Both heads insert at the proximal humerus lateral to the bicipital groove with the clavicular head more anterior and the sternocostal head more posterior [4,5]. Both heads are innervated by nerves originating from the brachial plexus [3,5-7]. The clavicular head is innervated by the lateral pectoral nerve, which is derived from the lateral cord, while the sternocostal head is innervated by the medial pectoral nerve, which is derived from the medial cord [3,5-7]. The main functions of the PM are flexion, adduction, and internal rotation of the humerus [1]. Although the PM is not of great importance for use in activities for daily living, it is an important muscle for individuals who enjoy physical and recreational activities. Injuries to the PM are a rare occurrence [8-10].

A systematic review by Elmaraghy and Devereaux in 2012 found 365 reported cases between 1822 and 2010, with 86 of those cases between 1822 and 1990 [11]. PM ruptures have been classified by Tietjen and modified by Bak et al. as type I (contusions), type II (partial ruptures), and type III (complete ruptures) [6]. Complete ruptures are further subdivided based on anatomic location as type III-A (muscle origin), type III-B (muscle belly), type III-C (musculotendinous rupture), type III-D (tendinous avulsion), type III-E (bony avulsion), and type III-F (tendon substance rupture) [6].

PM tendon ruptures typically occur in physically active males between the ages of 20 and 40 years old [10,12]. They may also occur in the elderly who are frail and when they are positioning, transferring, or dressing [13]. Most cases occur in athletes involved in weight lifting, wrestling, football, rugby, and windsurfing [7]. The most common activity or situation that causes PM tendon ruptures is through the “bench press” during eccentric contraction [14,15]. When patients have PM tendon ruptures, they often hear a pop along with a tearing sensation, followed by localized pain, swelling, ecchymosis, weakness, and a reduced range of motion around their affected shoulder [12,16].

Physical examination includes the asymmetry of their chest wall and axilla, weakness with internal rotation, and adduction [16,17]. A pectoral MRI has been shown to be the best imaging modality to confirm PM tendon ruptures [4,16]. In complete PM tendon ruptures, surgical treatment is superior compared to conservative plans for patients who are physically active as outcomes tend to be better based on patient satisfaction, strength, cosmesis, and return to preinjury activity [6,9,10,14,17,18]. According to a systematic review and meta-analysis by Gupton and Johnson, the two most successful repair techniques include the transosseous suture with the bone through technique and the suture anchor technique [10].

We present a case of a complete PM tendon rupture with a bony avulsion in an adolescent male who was initially misdiagnosed with biceps tendonitis. The purpose of this case report is to highlight the importance of having PM tendon ruptures in one’s differential diagnoses along with having a thorough history and physical examination because it is an uncommon injury [2].

## Case presentation

**Figure 1:**
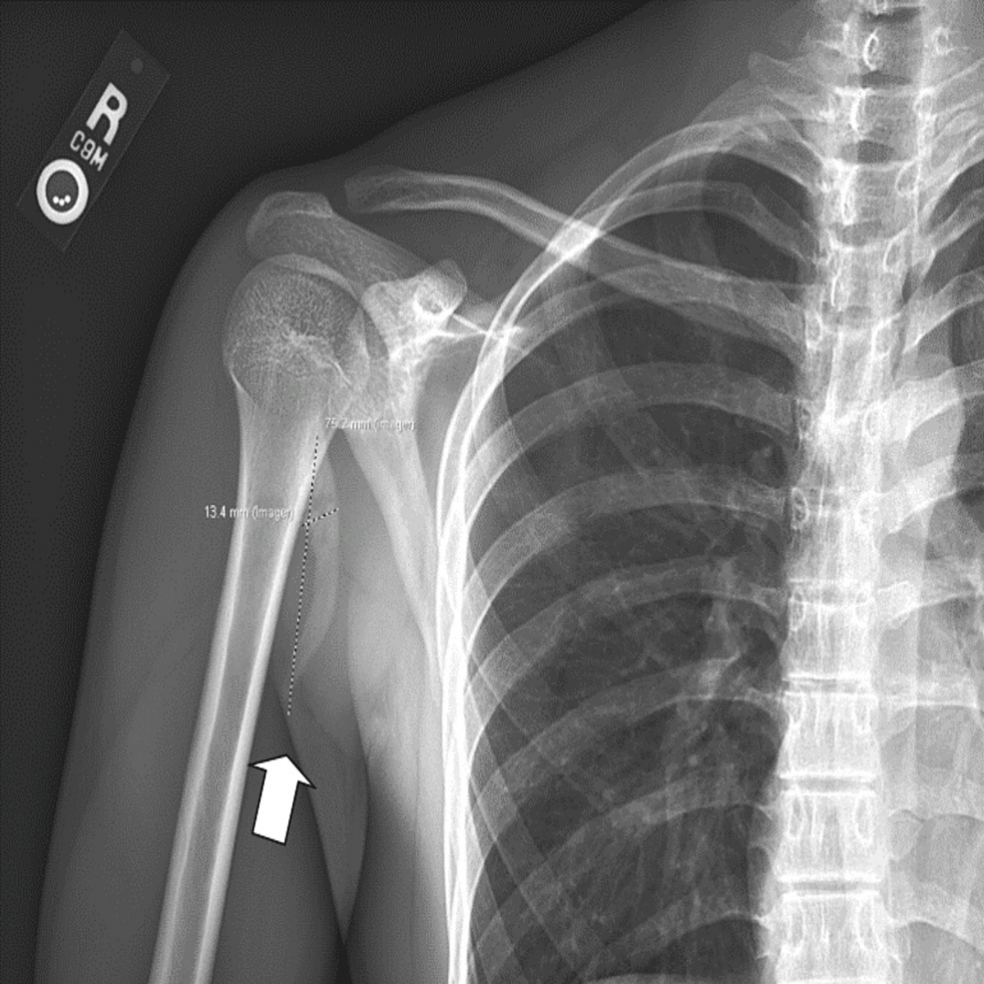
Right AP shoulder X-ray indicating heterotopic ossification at the proximal anteromedial shoulder measuring approximately 7.5 cm in length and 1.3 cm in thickness. The patient was diagnosed with biceps tendonitis at this time. AP: anteroposterior

A 16-year-old male presented to the urgent care clinic with right anterior shoulder pain that began two months prior and was seen by a primary care sports medicine physician. The patient stated that he landed on his right shoulder on a grass field while playing soccer. His pain was improving over time, but he re-aggravated his injury when he tried to throw a football, which prompted him to visit the urgent care clinic the following day. The patient states that he currently has moderate pain in his affected shoulder, which worsens with lifting and reaching and improves with rest. He denied neck pain, pain around his clavicle area, pain radiating down his arm, and arm numbness/paresthesia. The patient does not have a history of tobacco or alcohol use. The initial physical examination indicated tenderness at the right bicipital groove and pain with external rotation of the humerus. A right shoulder radiograph showed heterotopic ossification at the proximal anteromedial shoulder. This abnormal structure measured approximately 7.5 cm in length and 1.3 cm in thickness (Figure 1). The patient was diagnosed with biceps tendonitis at the time and was advised to ice the affected area for 20 minutes as needed for pain and take 400 mg of ibuprofen with breakfast and dinner for a week. A follow-up appointment with another sports medicine physician was scheduled.

At a follow-up appointment with the second clinician, the patient stated that he had intermittent stabbing anteromedial shoulder pain that was worse with lifting and better with rest. Upon physical examination, he was positive for the Speed and Yergason test. The patient had pain with resisting internal rotation and a scant painful arc, with no pain from resisting external rotation. He was negative for the drop arm, empty can, and full can test. His shoulder was freely mobile with a slight palpable fullness at the anterior shoulder that was suggestive of ossification at or near the proximal biceps tendon. The patient had no visible swelling or erythema and no palpable warmth. At this time, a shoulder MRI was ordered for further investigation.

**Figure 2:**
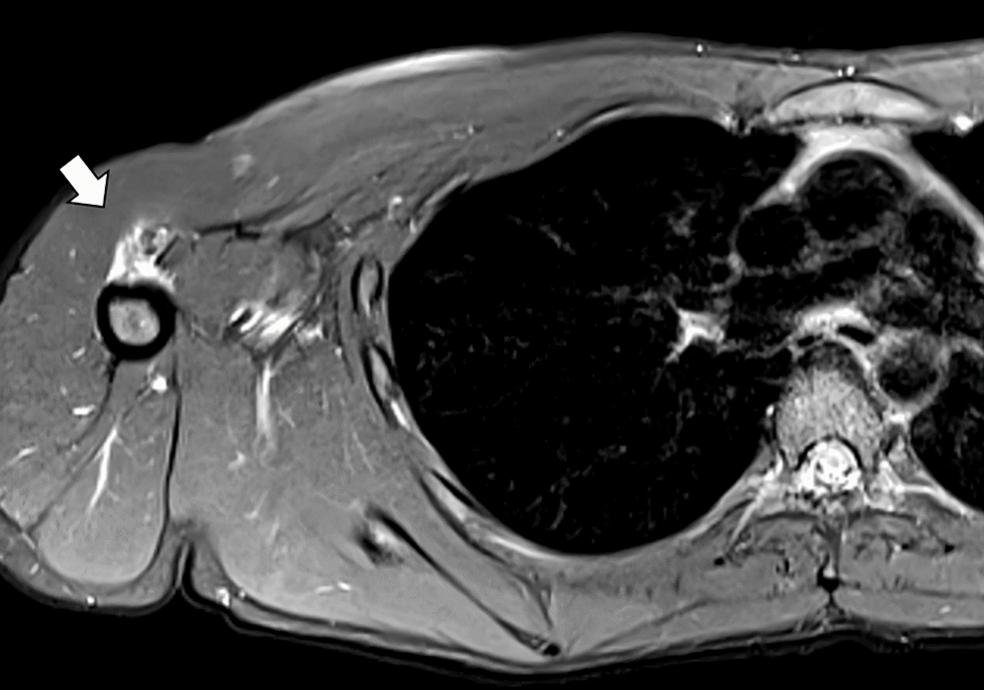
Right axial pectoral MRI indicating a bony fragment lateral to the biceps tendon with fluid at its insertion point.

**Figure 3:**
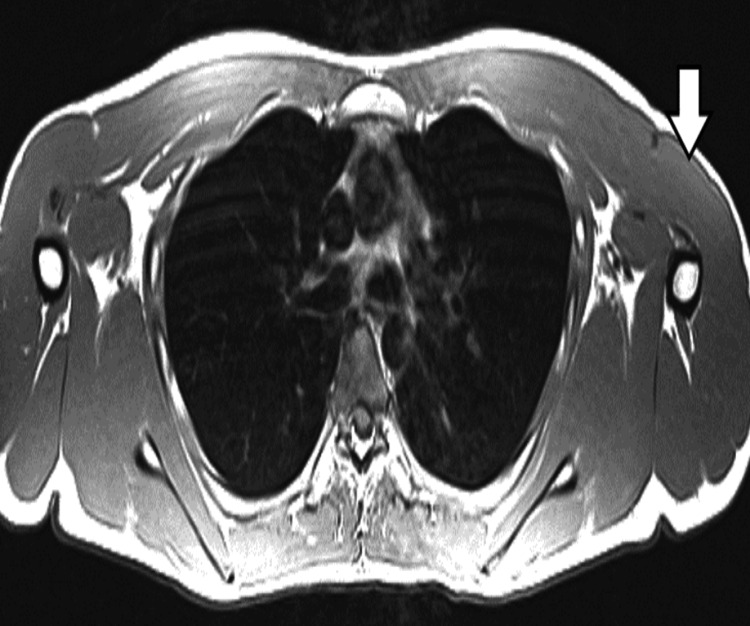
Axial pectoral MRI showing the contralateral (left) side with intact tendon and bony fragment adjacent to the right biceps with no tendinous attachment to the humerus.

A follow-up non-contrast MRI showed calcification at the proximal anteromedial shoulder, which led to suspicion that there was an injury to the pectoralis tendon. This led to the order of a pectoral MRI to clarify the severity of the injury and a consultation with an orthopedic surgeon (Figure 2). From the pectoral MRI, the right PM tendon is noted to be avulsed from its insertion on the humerus (Figure 2). There is a globular increased T2 signal lateral to the long head biceps tendon at the proximal shaft of the humerus. Mild edema is present. The pectoral MRI showed a complete rupture of the PM tendon with a 1 cm retraction and extensive calcification. An expanded view of the pectoral MRI shows the contralateral (left) side with an intact tendon (Figure 3).

Upon physical examination by the orthopedic surgeon, the patient was positive for a painful arc and had focal tenderness about the PM tendon insertion. The patient had a nearly full range of motion on his affected shoulder along with asymmetry to the axillary fold with the loss of the border and decreased PM tension compared to his left side. The patient was offered a PM tendon repair. Informed consent was obtained from the patient and family.

**Figure 4:**
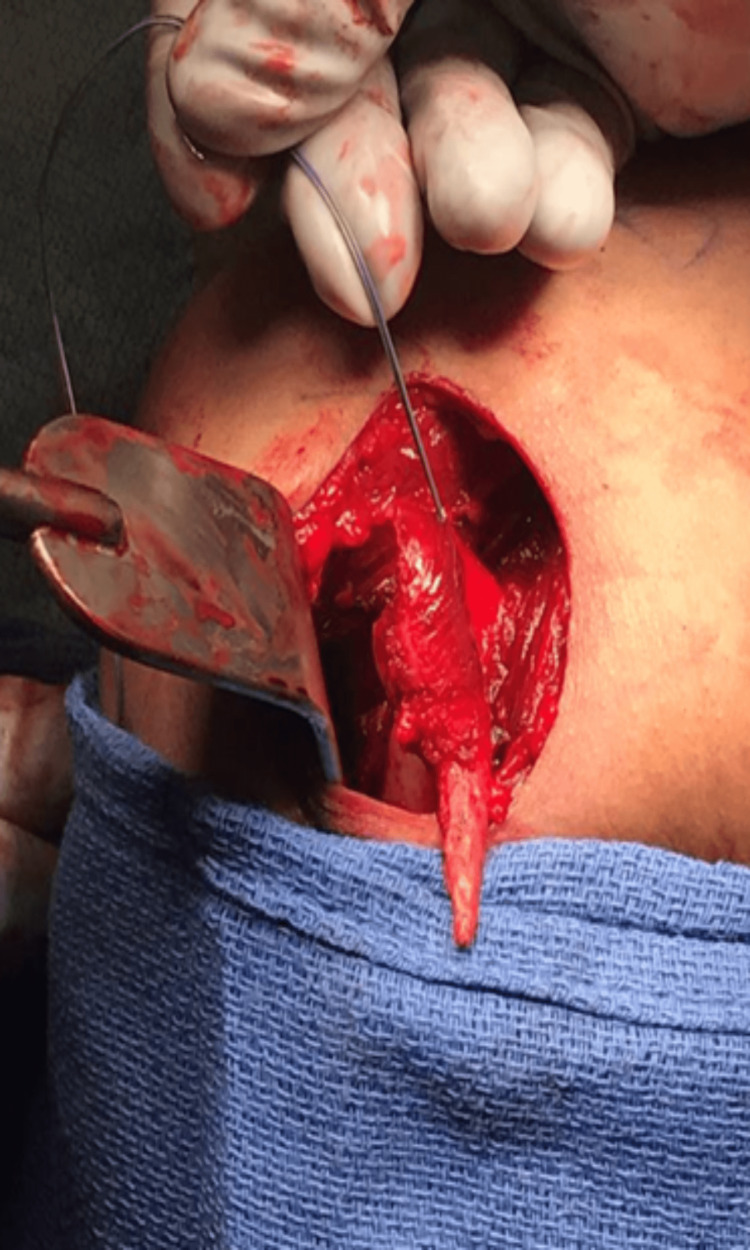
Bone mass attached to the pectoralis major.

**Figure 5:**
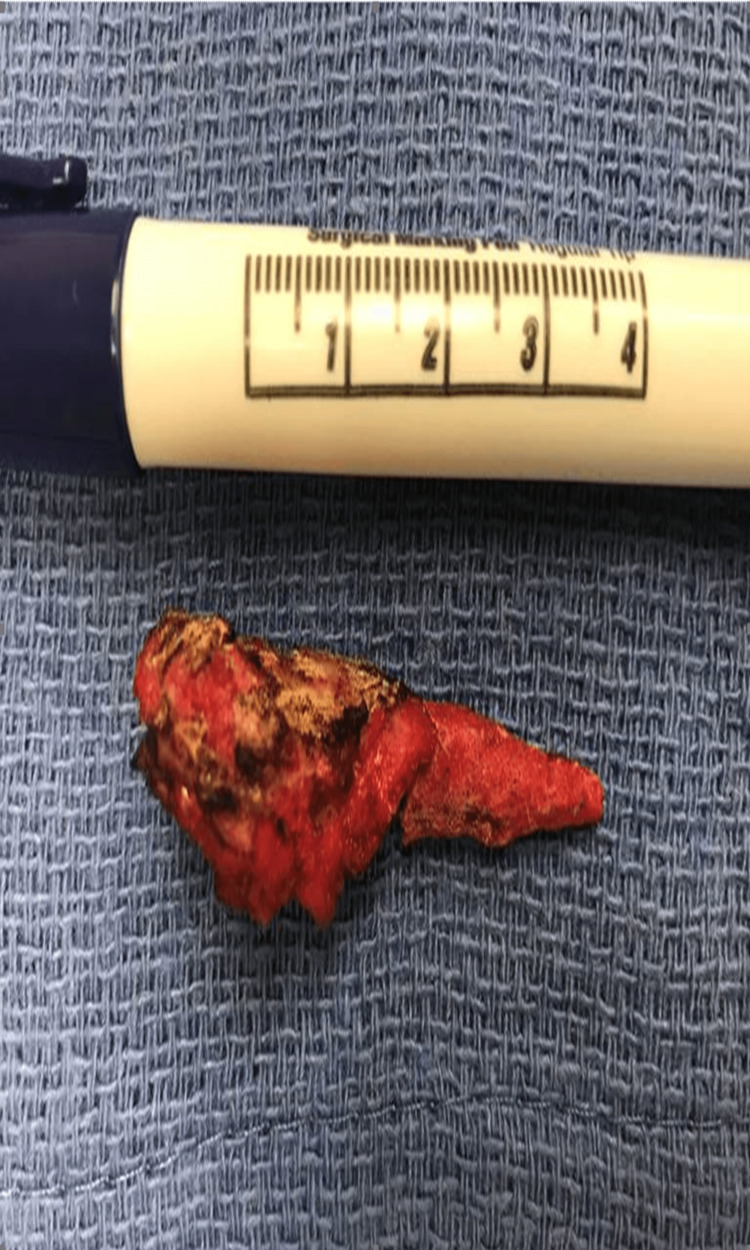
Four-centimeter resected bone avulsion.

The date of surgery was approximately between two and three months after the patient’s initial injury. During the surgical procedure, the patient was given intravenous cefazolin, and general anesthesia was administered. He was placed in a beach chair position. An open deltopectoral approach was performed at the right shoulder, and retractors were placed after the cephalic vein was identified. A large palpable bony mass was discovered just lateral to the biceps brachii with the PM tendon entirely attached to it (Figure 4). The bony fragment was liberated, and the humerus was prepared for repair with a rasp and Cobb elevator (Figure 5). The tendon was mobilized, and multiple traction sutures were placed. Three 3.5 mm Panalok suture anchors (DePuy Mitek, Raynham, MA) were placed, and the PM tendon was sutured in a Krackow fashion. One suture was used as a traction/reduction stitch on each anchor. They were all pulled to reduce the tendon, which seated nicely onto the humerus. They were then tied individually. One suture was tied to each anchor to further augment the repair. The arm was ranged, and a stable repair was obtained with a good establishment of the axillary fold. After the wound was irrigated, the incisions were closed with a 4-0 Biosyn suture (Medtronic, Minneapolis, MN); 2×2s and Tegaderm (3M, St. Paul, MN) were used for a sterile dressing, and the arm was protected with a sling.

**Figure 6:**
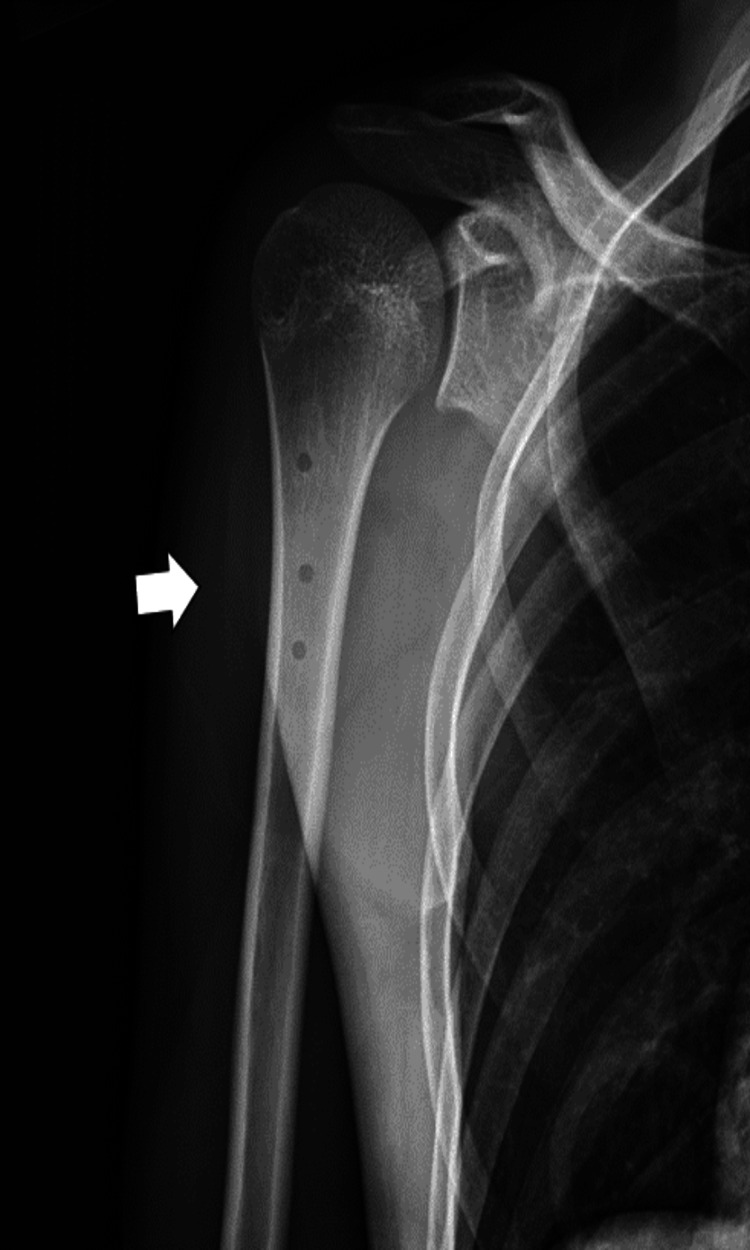
One week status post surgery of right AP shoulder X-ray indicating three lucenies in the right proximal humerus at the site of the suture anchors. The large, calcified body is no longer visible. AP: anteroposterior

At one week status post surgery, the patient was making excellent progress. His pain was well controlled, and he denied any concerns. The patient’s wounds were well healed without the evidence of erythema fluctuance or drainage. His circulation, motor, and sensation were intact. Range of motion was limited as expected, and there was no evidence of infection. The patient was referred to a physical therapy program along with a follow-up in four to six weeks. The program had five phases that involved immobilization for the first three weeks, followed by a gradual increase in the range of motion and strength exercises with a return to full activity between five and seven months. The patient’s shoulder X-ray showed that the large, calcified body is no longer visible (Figure 6). There were three lucencies in the right proximal humerus at the site of the suture anchors. The remainder of the shoulder is normal, alignment is anatomic, and no radiopaque foreign bodies were noted. At six weeks status post surgery, the patient still had excellent progress without complications, and his pain was well controlled. He still had no evidence of erythema, fluctuance, or drainage. His range of motion was progressing as expected, and there was no evidence of infection. The subsequent plan included the continuation of physical therapy with another follow-up appointment in six weeks; however, the patient did not attend future appointments.

## Discussion

PM tendon ruptures are a rare occurrence, typically seen in physically active males between the ages of 20 and 40 years old while doing bench presses [2,12]. Historically, PM tendon ruptures were worked-related injuries, but most PM tendon ruptures have occurred within the past two decades due to a more physically active population [8,17]. Due to the rarity of PM tendon ruptures, it is possible that clinicians might misdiagnose this condition [9,14]. To the authors’ knowledge, there are no previously reported cases of PM tendon ruptures with an avulsion fracture from soccer players in the adolescent demographic. Although the PM is not required for activities of daily living, it is needed to participate in many physical and recreational activities [4,6,7]. It is important for clinicians to include PM tendon ruptures in their differential diagnosis so that patients with this condition can promptly receive appropriate surgical interventions, return to their recreational activities, and maintain their quality of life [9].

Diagnosis begins with a thorough history. Patients often recall feeling a pop and tearing sensation in their affected shoulder, followed by pain and weakness [4,6,19]. Upon physical examination, swelling and ecchymosis might be present at their anterior chest or axilla and down their arm [9,10]. It is important to compare the uninjured side as there can be visible deformity and loss of symmetry at the axilla fold [4,5]. Tenderness may be noted along the insertion point of the PM at the axillary fold in addition to pain and weakness with adduction and internal rotation of the humerus [17,18].

According to Chiavaras et al., a pectoral MRI is the preferred imaging modality to diagnose a PM tendon rupture. In acute PM tendon ruptures, there will be abnormal T2 signal intensity at the location of the injury. A PM tendon rupture that produces a bony avulsion would show the bone fragment with surrounding hemorrhage and edema [2]. The treatment plan for PM tears at the origin or muscle belly typically involves rest and physical therapy [2,14]. However, more distal PM tears at the musculotendinous junction, distal tendon, or humeral attachment require surgical intervention [2,14]. In cases where a patient has a type III PM tendon rupture, surgical intervention within the first six weeks of the injury is optimal. Chronic PM tendon tears can present with challenges such as muscle atrophy and retraction, scar formation, altered anatomy, and increased risk of having poor-quality or nonviable tendon tissue [6,18]. These tears might require tendon reconstruction with autograft or allograft, which is why the early diagnosis of a PM tendon rupture is important so that surgical intervention can begin [2,16].

There have not been many studies that discuss the rehabilitation process for post-operative PM tendon repairs. In an article written by Manske and Prohaska, the process of post-operative rehabilitation for PM tendon ruptures involves the gradual restoration of one’s full functional range of motion and strength with the ultimate goal of having the patients return to their preferred activities [20]. They describe a four-phase rehabilitation process with the first phase involving immobilization for two weeks, followed by a gradual increase in the range of motion and strength exercises. For the patients to return to their activity, they must be pain-free, have full or adequate range of motion, and have normal strength.

## Conclusions

PM tendon ruptures are rare, especially in the adolescent population. Many clinicians might not include it in their differential diagnosis. This case highlights the importance of having a thorough history and physical examination to prevent a misdiagnosis. Early surgical interventions are more beneficial than conservative plans for PM tendon ruptures. A pectoral MRI is the preferred imaging modality in one’s diagnostic workup. Patients would benefit from having surgery within six weeks of the injury to avoid complications such as atrophy, retraction, and scar formation seen in chronic PM tendon ruptures. For patients who have active lifestyles, early surgical interventions will allow them to return to their activities and maintain their quality of life.
